# Supplementary material for: A set of multi-entry identification keys to African frugivorous flies (Diptera, Tephritidae)
Source: Zookeys. 2014 Jul 24;(428):97–108. doi: 10.3897/zookeys.428.7366 (PMC4143993; doi:10.3897/zookeys.428.7366)
Supplement: Supplementary material 6 — Key to Ceratitis [file zookeys-428-097-s006.zip › SF6_ZooKeys_key to Ceratitis/key/SF6_ZooKeys_key to Ceratitis/Media/Html/Ceratitis stipula.htm]

Ceratitis (Pterandrus) stipula, n


***Ceratitis*** **(*Pterandrus*) *stipula* De Meyer & Freidberg**

Body
length: 4.60 (4.05-5.00) mm; wing length: 4.98 (4.20-5.30) mm.

Male

Head: Antenna yellow to orange. First flagellomere 2-3 times
as long as pedicel. Arista with short rays; ventral rays shorter and sparser
than dorsal rays, especially basally. Frons yellow, sometimes with dark orange
patches; with short scattered setulae of same color as frons. Frontal setae
well developed, sometimes slightly underdeveloped. Face yellowish white, gena
sometimes darker yellow. Genal seta and setulae black, well developed.

Thorax: Postpronotal
lobe yellowish white; without spot. Scutal pattern, ash-gray ground color; with
streaks and darker markings but without distinct spots or clearly defined
stripes except prescutellar white separate markings, with pale area in between.
Scapular setae dark. Scutellum yellowish white, basally without spots
(sometimes darker coloration posterior of prescutellar markings but not
distinct spots), apically with three separate dark spots, extending to basal
half. Anepisternum along ventral margin yellowish brown; setulae pale.

Legs:
Yellow except where otherwise noted; setation typical for subgenus, mainly
dark. Foreleg: femur brown; posteriorly with poorly developed bush of dispersed
long dark setulae along entire length, posterodorsal setulae longer, especially
in apical 0.33, posteroventral setulae sometimes pale brown; ventral setae
dark. Midleg: femur largely brown except distally pale, dorsally pale part
extending further basally; ventrally with dark feathering along distal half;
anteroventrally with short but conspicuous dark setulae except in pale part;
tibia broadened; largely yellowish brown with extremities pale, with weak
silvery shine when viewed from certain angle; with black feathering dorsally
along distal 0.9 and ventrally along distal 0.6, anteriorly with short to
moderately long setulae, second row with shorter feathering present anterior to
dorsal feathering. Hindleg: femur dark brown except distally; at apical 0.25
with longer setulae dorsally and ventrally.

Wing:
banding brown. Interruption between marginal and discal bands near vein
R_1_ clear and complete; cubital band free; medial band absent;
crossvein R-M at or just proximal to middle of discal cell. Apex of vein R_1_
distal to level of crossvein R-M. Crossvein DM-Cu variable.

Abdomen:
Mostly yellow. Fusion area between tergites 1 and 2 with orange-brown patches.
Tergites 2 and 4 with pale gray band occupying almost entire tergite , at most
narrowly yellow anteriorly. Tergite 3 with distinct brownish black transverse
band along� posterior half. Tergite 5 anteriorly with two brown spots,
posterior margin narrowly brownish. Male epandrium in lateral view with
posterior lobe of lateral surstylus short and straight, anterior lobe
pronounced.

Female

As
male except for the following characters. Legs without feathering; femora dark
yellow, sometimes with brownish streaks, forefemur sometimes largely brownish,
posteroventrally with dark setulae. Oviscape shorter than preabdomen. Aculeus
about seven times longer than wide; tip with distinct apical indentation,
narrow and lateral margin strongly sinuous.

(Description
after De Meyer & Freidberg, 2006)
